# Rethinking Recovery: Psychological Flexibility and Growth Across Diverse Recovery Approaches

**DOI:** 10.3390/bs16030366

**Published:** 2026-03-05

**Authors:** Nicole M. Pyke, David R. Perkins, Emily K. Sandoz

**Affiliations:** 1 Department of Psychology, Tulane University, New Orleans, LA 70118, USA; 2 Department of Psychology, University of Louisiana, Lafayette, LA 70503, USA; david.perkins@louisiana.edu; 3 University Honors Program, University of Louisiana, Lafayette, LA 70503, USA; emily.sandoz@louisiana.edu

**Keywords:** recovery capital, substance use recovery, psychological flexibility, posttraumatic growth, abstinence, trauma

## Abstract

Although recovery capital and psychological flexibility have each been associated with substance use recovery, their combined role in fostering posttraumatic growth remains unclear. This study integrates recovery capital theory with psychological flexibility and posttraumatic growth frameworks to test a theory-driven model of adaptive recovery processes. The study sought to (1) examine whether recovery capital or abstinence predicts posttraumatic growth, (2) assess whether recovery capital or abstinence status predicts psychological flexibility, and (3) test whether psychological flexibility statistically mediates the relationship between recovery and posttraumatic growth. A national sample of 244 adults in recovery from substance use disorder completed the Assessment of Recovery Capital, the Personalized Psychological Flexibility Index, and the Posttraumatic Growth Inventory—Expanded. Multiple linear regressions addressed the first two aims. For Aim 3, the mediation model was tested using the SPSS PROCESS macro (version 4.0) with 5000 bootstrap resamples. Recovery capital significantly predicted both posttraumatic growth and psychological flexibility. Abstinence status did not predict posttraumatic or psychological flexibility. Psychological flexibility partially mediated the association between recovery capital and posttraumatic growth. Psychological flexibility may be a key mechanism by which recovery capital fosters posttraumatic growth, supporting interventions that enhance both resources beyond abstinence alone.

## 1. Introduction

In 2024, nearly 48.4 million people in the United States met the criteria for a substance use disorder in the past year, while only 12.3% ever received treatment (Substance Abuse and Mental Health Services Administration, 2024). Standard treatment traditionally emphasizes *abstinence*, the discontinuation of harmful substance use as the primary goal (Beaugard et al., 2025; Meadows et al., 2024). The eliminative approach suggests that a sustained five-year abstinence may be necessary for healthy outcomes (DuPont et al., 2015; Kelly et al., 2019), yet growing evidence suggests abstinence alone may be insufficient. Despite brief successes, long-term abstinence is complex for many individuals (Cioffi et al., 2024; Witkiewitz & Tucker, 2025), requiring ongoing maintenance throughout one’s lifetime (Dennis et al., 2024; Goshorn et al., 2023). From this shift in perspective emerges a pathway toward harm reduction principles, meeting the individual where they are at to support multiple pathways aligned with individuals’ goals and well-being (O’Sullivan et al., 2025). In this way, recovery can be conceptualized as the extent to which a person who has engaged in problematic substance use considers themselves to be in recovery (Brophy et al., 2023; Kelly et al., 2018a; Witkiewitz, 2020; Zemore et al., 2023). National surveys have increasingly recognized self-identified recovery as a meaningful indicator of abstinence status (Eddie et al., 2022; Jones et al., 2020; Pasman et al., 2025). Recovery identity is also shaped by social context and stigma, influencing whether a person discloses recovery status and access to support (Lancaster et al., 2025). The broader perspective moves beyond a singular focus on substance cessation toward a more holistic understanding of what supports lasting well-being.

### 1.1. Recovery Capital

These addiction recovery strengths have been conceptualized as *recovery capital* (RC), the internal and external resources a recovered person utilizes in advancing toward positive outcomes (Best et al., 2026; Cloud & Granfield, 2008). RC encompasses dimensions such as social support, housing, psychological health, coping resources and community involvement. Recent reviews have confirmed its utility as a framework for monitoring these strengths that support well-being beyond symptom reduction (Best et al., 2026; Bunaciu et al., 2024; Day et al., 2025). However, many biopsychosocial factors complicate pathways to recovery. Among the most prominent factors is a history of trauma (Karsberg et al., 2025; Leri et al., 2026; Patel et al., 2025). Adverse childhood experiences are common among those in substance use treatment (Leza et al., 2024; Poulsen et al., 2024) and are associated with elevated risk for psychiatric disorders, including anxiety and mood disorders (Daníelsdóttir et al., 2024; Grummitt et al., 2024), sometimes with marked psychological impairment. Coping responses to trauma vary—individuals with greater pre-trauma resilience tend to adopt more approach-oriented strategies (Scoglio et al., 2024). These varied coping pathways suggest engagement with adversity may be as important as the resources they accumulate.

### 1.2. Posttraumatic Growth

Posttraumatic growth (PTG) is the experience of positive intrapersonal and interpersonal changes following traumatic life events (Dell’Osso et al., 2022; Tedeschi & Calhoun, 1995). PTG can involve: (1) improved sense of self, (2) greater connectedness to self and others, (3) openness to and awareness of new possibilities, (4) life appreciation and gratitude and valued living, and (5) spiritual openness, a sense of connection that is greater than self (Dell’Osso et al., 2023; Tedeschi et al., 2018). In this way, both trauma and substance use may be ultimately associated with positive factors that contribute to quality-of-life improvements and growth.

### 1.3. Recovery and Posttraumatic Growth

Addiction-related growth may be related to the positive life changes involved in recovery (Runyan et al., 2024), and recent evidence further strengthens this connection (Hutto et al., 2024). Recovery capital, particularly social support through mutual-aid groups, has shown to predict PTG better than abstinence alone (Haroosh & Freedman, 2017), consistent with findings that recovery resources may co-occur with growth over time (Härd et al., 2022; Runyan et al., 2024) and with increased meaning and spirituality (Chukwuorji et al., 2025; Ibrahim et al., 2022). However, Haroosh and Freedman’s (2017) study remains the only one to directly model recovery capital components as predictors of PTG among individuals with substance use disorders, and no subsequent study has directly replicated the modeling of RC and abstinence on PTG, a gap confirmed through a targeted literature review.

Although direct investigations of the RC–PTG link remain limited, emerging work offers insight, including identity transformation, social integration, and resilience building, which parallels the condition theorized to foster posttraumatic growth (Jean-Berluche, 2025). Recovery identity has been shown to buffer the negative effects of trauma on recovery capital in collegiate recovery populations (Francis et al., 2025). Theoretical advances further suggest that community-level recovery resources may create a cascading effect on personal and social capital (Irish et al., 2025), suggesting that mechanisms by which RC operates extend beyond individual assets. Taken together, these findings offer recovery not only as an abstinence focus, but the accumulation of dynamic resources which may set the stage for positive transformation. What remains unclear is the process by which these resources translate into growth. One potentially relevant process is psychological flexibility.

### 1.4. Psychological Flexibility

Psychological flexibility (PF) is the capacity for adaptive and meaningful action despite distressing thoughts, feelings, and/or sensations (S. C. Hayes et al., 2006, 2011; Malo et al., 2024). PF is the purported mechanism of Acceptance and Commitment Therapy (ACT), which aims to facilitate valued living by undermining rule-based avoidance (S. C. Hayes et al., 2006; Levin et al., 2024). Three components comprise PF: (1) openly experiencing distress, (2) strategic handling of distressing thoughts, feelings, and sensations, and (3) taking action to facilitate individual goals and values (Cherry et al., 2021; Kashdan et al., 2020; Macri & Rogge, 2024). Greater well-being has been consistently linked with higher PF across diverse populations (Rutschmann et al., 2024), and workplace contexts (Archer et al., 2024).

### 1.5. Psychological Flexibility and Recovery

PF-based approaches for substance use disorders include mindfulness-based intervention, which reduces craving and promotes awareness. Greater PF is associated with reduced problematic substance use in veterans (Reilly et al., 2023), enhanced coping with cravings and improved abstinence rates (Santiago-Torres et al., 2024), and committed action toward recovery goals (Hooker et al., 2024). Recent evidence also illustrates that PF mediates the relationship between distress tolerance and perceived stress among individuals with substance use disorder (Yıldız & Büyükfırat, 2025), and that PF and self-compassion jointly account for reduced substance misuse and improved well-being (Arslan et al., 2024). A recent systematic review and meta-analysis also linked psychological inflexibility (including experiential avoidance) with greater substance use severity and lower outcomes (Barrado-Moreno et al., 2025). Although these findings link PF to specific recovery outcomes, the literature has not directly explored PF in relation to more expansive indicators of recovery, such as recovery capital. As PF increases, it can significantly broaden behavioral repertoires in contexts that evoke distress and trauma-related experiences (Bonanno et al., 2023; Butts & Gutierrez, 2018).

A growing body of evidence also links PF to PTG. Although attaining PF after trauma can be difficult (Bruno et al., 2024), higher PF has been shown to strengthen the relationship between post-traumatic stress and PTG in trauma populations (Türk, 2024). PTG is also associated with facets of PF such as mindfulness and self-compassion (Chen et al., 2024; Wong et al., 2025), and work demonstrates that PF partially mediates the relationship between adverse childhood experiences and PTG (Sachdeva & Shukla, 2024) and between PF and PTG through active coping in cancer patients (Akcan et al., 2024). Together, these findings position PF as a transdiagnostic process relevant to both recovery from substance use and trauma-related growth.

Conceptually, PF may serve as a process-level mechanism linking recovery capital to posttraumatic growth. RC provides the resources and conditions that support recovery—social support, coping skills, community involvement—but resources alone do not automatically translate to growth outcomes. PF, with its emphasis on open engagement with difficult internal experiences, present moment awareness, and value-directed action, represents the behavioral process through which individuals may transform to approach resources into meaningful action and life experiences. Specifically, PF may enable individuals to approach rather than avoid trauma-related distress, to flexibly apply recovery resources in service of personal values, and to engage in deliberate cognitive processing that characterizes PTG (Sachdeva & Shukla, 2024; Türk, 2024).

### 1.6. Current Study

Although recovery capital, psychological flexibility, and posttraumatic growth have each received empirical investigation in the context of substance use recovery, these constructs have largely been examined in isolation or association. No prior study has directly compared recovery capital and abstinence as predictors of posttraumatic growth while simultaneously examining psychological flexibility as a mediator of these associations among individuals with co-occurring trauma and substance use histories. The study addresses these gaps using a nationally recruited sample of adults endorsing diverse recovery pathways—including both abstinent and non-abstinent approaches—which extends the evidence beyond single-site treatment-specific designs that characterize much of the literature.

The study sought to examine the relationships between recovery types (recovery capital and abstinence), psychological flexibility, and posttraumatic growth among individuals with histories of trauma and problematic substance use. Recovery is defined broadly to include improvements in well-being and daily functioning after problematic substance use, encompassing multiple dimensions beyond abstinence (Kelly et al., 2018b; Witkiewitz et al., 2022). We distinguish between (a) recovery status, operationalized as current adherence to abstinence, and (b) recovery capital, operationalized as a total multidimensional score of resources. Specifically, we aimed to (1) examine whether recovery capital and abstinence were associated with posttraumatic growth, (2) examine whether recovery capital and abstinence were associated with psychological flexibility, and (3) test whether psychological flexibility mediated the association between recovery capital and posttraumatic growth. While meeting this third aim involved the use of a mediation framework, we interpreted these results as statistical associations only, without assuming causation or temporal ordering.

Hypotheses were as follows: (H1) recovery capital would be positively associated with psychological flexibility; (H2) recovery capital would be positively associated with posttraumatic growth; and (H3) psychological flexibility would mediate the relationship between recovery capital and posttraumatic growth. Given the mixed evidence for abstinence-only indicators in broader functioning, abstinence status was examined as an exploratory predictor.

## 2. Methods

### 2.1. Participants

Participants were recruited via CloudResearch Connect (Chandler et al., 2019; Douglas et al., 2023), an online crowdsourcing platform that facilitates access to a national sample. Eligible participants were adults (18+) residing in the U.S. who reported a history of problematic substance use and either currently self-identified as being in recovery or were actively working toward recovery. Of 366 individuals who initiated the survey, 122 were excluded from the analysis due to missing data on one or more variables required for the primary analyses (listwise deletion in SPSS). The final analytic sample comprised 244 participants (62.30% male, 36.97% female; *M* age = 35.91, *SD* = 10.32). Within this sample, 60.66% reported abstinence-based recovery and 39.34% reported non-abstinence-based recovery approaches. Approximately 65.98% self-identified as being in recovery (n = 161), while 34.02% (n = 83) reported substance use histories but did not endorse a recovery identity. See Table 1 for further demographic and recovery characteristics.

### 2.2. Measures

Demographics Questionnaire: A brief questionnaire gathered self-report data on demographics, including age, gender, race, recovery status, mutual-aid participation, time since last use, spirituality, and religious affiliation. To operationalize self-identified recovery, the present study adopted two items from the National Survey on Drug Use and Health (NSDUH) in 2018, which recognized the importance of recovery identity (Jones et al., 2020): (1) “Do you think you ever had a problem with your own drug or alcohol use?,” and (2) “At this time, do you consider yourself to be in recovery or recovered from your own problem with drug or alcohol use?”. Item 1 served as an eligibility screener, and Item 2 was included in the demographic questionnaire to confirm recovery identity (see Table 1: “Recovery Identity”).

Life Events Checklist for DSM-5 Standard Version (LEC-5). The LEC-5 Standard Version (Weathers et al., 2013) is a brief 17-item self-report measure of an individual’s trauma experience throughout their lifespan. Participants rate the items from varying levels of trauma exposure on a nominal 6-point scale. Sample items include levels assessed from: “Happened to me,” “Witnessed it,” “Learned about it,” “Not sure,” and “Does not apply.” Test–retest reliability has been reported in undergraduate samples at 0.62 to 0.64 for direct trauma exposure (Pugach et al., 2021). When paired with the PTSD Checklist (PCL-5), the measure had good internal consistency (α = 0.86), supporting the LEC-5’s utility as a trauma screening measure (Cao-Noya & Benuto, 2025), which was demonstrated in the current study (α = 0.85).

The Posttraumatic Growth Inventory Expanded (PTGI-X). The PTGI-X (Tedeschi et al., 2017) includes 25 items rated on a 6-point Likert-type scale, with responses from 0 = “I did not experience this change as a result of my crisis” to 5 = “I experienced this change to a great degree as a result of my crisis.” Items in the PTGI-X were amended to replace “crisis” with “these events” to refer to the events from the LEC-5, completed just before the PTGI-X. The PTGI-X shows good convergent and discriminatory validity, internal reliability overall (α = 0.97), and improved internal reliability for improved SC to SEC factors (α = 0.83–0.91) (Tedeschi et al., 2017). Recent validation has supported the structural validity of PTGI-X adaptations in clinical samples (Ascencio Huertas et al., 2025). Items were totaled to create a composite score (0–125), with higher scores indicating a more significant growth experience. The measure showed good internal consistency (α = 0.93).

Assessment of Recovery Capital (ARC). The ARC (Groshkova et al., 2013) is a 50-item self-report assessment measuring recovery capital in five domains (Bunaciu et al., 2024). ARC total score was computed by summing items across subscales. The overall ARC measure showed good internal consistency (α = 0.86). Abstinence status was operationalized as a binary indicator derived from two items on the ARC Substance Use & Sobriety subscale: “I am currently completely sober” and “I have had no recent periods of substance intoxication.” Participants endorsing both items were coded abstinent = 1; all others were coded non-abstinent = 0. This study-defined binary abstinence variable (ARC-SUR) was not included in the total ARC score.

The Personalized Psychological Flexibility Index (PPFI): The PPFI (Kashdan et al., 2020) is a 19-item self-report assessment of psychological flexibility, unique among PF scales due to the addition of a qualitative goal that is identified and reflected upon throughout. For example, relating to an expansive recovery goal, a participant would rate the statement “This goal is central to my life” based on its relation to personal recovery goals. The PPFI utilizes a 7-point Likert scale, 1 = Strongly Disagree, through to 7 = Strongly Agree. The PPFI shows good internal consistency across all samples (α = 0.77–0.87). The measure showed good internal consistency (α = 0.82).

### 2.3. Procedures

After providing informed consent, participants completed self-report questionnaires via an online survey on the CloudResearch Connect platform. The survey explained the purpose of the study, potential risks, benefits, compensation, and participants’ rights, including voluntary withdrawal. Those who completed all measures received $4 USD. Study procedures were approved by the Institutional Review Board (IRB # IRB-23-125-PSYC-OL) at the university from which the research was conducted.

### 2.4. Statistical Analyses

Data were cleaned, coded, and analyzed using IBM SPSS Statistics (Version 31; IBM Corp., Armonk, NY, USA) software. Screening procedures addressed missing data, univariate outliers, and rapid completions (<5 min). Descriptive statistics were calculated for demographics, recovery characteristics and primary study variables. Internal consistency of all measures (ARC, PPFI, PTGI-X, LEC-5) was assessed using Cronbach’s alpha. Bivariate correlations examined preliminary relationships among key variables and primary models utilized listwise deletion in analyses.

A priori power analysis: Before data collection, we used G*Power 3.1 (Faul et al., 2007; Kang & Huh, 2021) to estimate sample size for multiple regression with two predictors (α = 0.05, f^2^ = 0.15). Results indicated N = 107 would provide 80% power to detect a moderate effect in the regression models. G*Power does not directly estimate power for bootstrap indirect effects; therefore, we set a target of N ≈ 250 to (a) allow up to 20% attrition/exclusions/bots and (b) retain precision for subgroup and potential for sensitivity analyses. The final N = 244 exceeded the regression power target.

To test the study hypotheses, a series of linear regression models corresponding to each study aim was constructed. For Aim 1, simple linear regressions were used to separately examine recovery capital and abstinence status as concurrent predictors of posttraumatic growth, followed by a simultaneous multiple regression model including both predictors to determine their relative contributions. For Aim 2, similar simple regressions were conducted to evaluate recovery capital and abstinence status as concurrent predictors of psychological flexibility.

For Aim 3, we examined whether psychological flexibility might mediate the observed relationship between recovery capital and posttraumatic growth. To explore this question, we conducted a mediation using the SPSS PROCESS macro (Version 4.0; A. F. Hayes, 2022), with recovery capital entered as the predictor variable, psychological flexibility as the mediator variable, and posttraumatic growth as the outcome. Indirect effects were estimated using 5000 bootstrap samples, and all results reported standardized coefficients (z-scores), standard errors (SE), exact *p*-values, 95% confidence intervals, and R_2_. A significance level of *p* < 0.05 (two-tailed) was used. As sensitivity analyses, models were re-estimated (a) controlling for recovery characteristic “sobriety time,” an ordinal, categorical measure ranging from less than 3 months to more than 20 years, with higher score indicating longer “time since last use,” and (b) using an ARC total score excluding the two sobriety items (ARC-SUR). We relied on bootstrapped confidence intervals to improve the indirect effects (Alfons & Schley, 2025).

## 3. Results

Table 1 summarizes participant demographic and recovery characteristics. As illustrated in Table 2, posttraumatic growth (PTGI-X) was positively correlated with recovery capital (ARC; *r* = 0.21, *p* < 0.001), psychological flexibility (PPFI; *r* = 0.26, *p* < 0.001), recovery importance (*r* = 0.43, *p* < 0.001), and spirituality importance (*r* = 0.47, *p* < 0.001). Recovery capital was positively correlated with abstinence status (ARC-SUR; *r* = 0.44, *p* < 0.001) and psychological flexibility (*r* = 0.19, *p* < 0.01). Abstinence status was not significantly correlated with PTGI-X or PPFI but was positively correlated with recovery importance (*r* = 0.16, *p* < 0.05).

Table 3 reports regression models for Aims 1 and 2. For Aim 1, regression analyses examined which recovery indicators were most strongly associated with posttraumatic growth (PTGI-X). As shown (Model 1a), recovery capital (ARC) significantly predicted PTGI-X (*b* = 1.09, *SE* = 0.31, *t* = 3.42, *p* < 0.001, 95% *CI* [0.46, 1.71], *R*^2^ = 0.046), whereas abstinence status (ARC-SUR) was not a significant predictor (Model 1b: *b* = 2.56, *SE* = 3.98, *t* = 0.64, *p* = 0.521). When both predictors were entered simultaneously (Model 1c), ARC remained significant (*b* = 1.24, *SE* = 0.35, *t* = 3.49, *p* < 0.001), and abstinence status remained non-significant (*b* = −4.09, *SE* = 4.30, *t* = −0.94, *p* = 0.351; *R*^2^ = 0.05). For Aim 2, similar models examined associations with psychological flexibility (PPFI). ARC significantly predicted PPFI (Model 2a: *b* = 0.34, *SE* = 0.11, *t* = 2.94, *p* = 0.001, 95% *CI* [0.11, 0.57], *R*^2^ = 0.035), whereas abstinence status was not significant (Model 2b: *b* = 1.14, *SE* = 1.45, *t* = 0.79, *p* = 0.431). In the simultaneous model (Model 2c), ARC remained significant (*b* = 0.37, *SE* = 0.13, *t* = 2.88, *p* = 0.001) and abstinence status remained non-significant (*b* = −0.87, *SE* = 1.59, *t* = −0.54, *p* = 0.591; *R*^2^ = 0.036). Given the non-significant associations for abstinence status, the mediation model focused on recovery capital (ARC).

**Figure 1:**
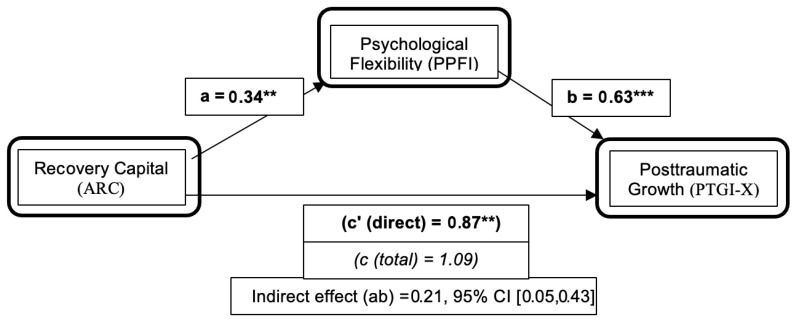
The mediating effect of psychological flexibility on the relationship between recovery capital and posttraumatic growth (N = 244). ** *p* < 0.01, and *** *p* < 0.001. Note: PTGI-X = Post-Traumatic Growth Inventory-X; ARC = Assessment of Recovery Capital; PPFI = Personalized Psychological Flexibility Index.

For Aim 3 (H3), we tested whether psychological flexibility (PPFI) statistically accounted for part of the association between recovery capital (ARC) and posttraumatic growth (PTGI-X). Using PROCESS with 5000 bootstrap resamples, the indirect effect was significant (indirect effect = 0.21; 95% *CI* [0.03, 0.34]), consistent with partial mediation. ARC was positively associated with PPFI (a path: *b* = 0.34, *SE* = 0.11, *p* = 0.001), and PPFI was positively associated with PTGI-X, controlling for ARC (b path: *b* = 0.63, *SE* = 0.17, *p* < 0.001). When PPFI was included in the model, the direct association between ARC and PTGI-X was reduced but remained significant (direct effect: *b* = 0.87, *SE* = 0.32, *p* = 0.006; see Table 3 and Figure 1). Sensitivity analyses controlling for time since last use and re-estimating models using an ARC total score excluding the two sobriety items yielded the same pattern of findings.

## 4. Discussion

The present study advances recovery theory by integrating recovery capital, psychological flexibility, and posttraumatic growth into a unified model of adaptive recovery. Findings suggest that recovery capital, rather than abstinence status, is a more robust predictor of posttraumatic growth. Prior to the present study, Haroosh and Freedman (2017) provided the most direct examination of recovery capital components as predictors of posttraumatic growth among individuals with a history of substance use disorder, finding that social support, not abstinence, accounted for significant variance in growth. Runyan et al. (2024) extended this by demonstrating real-time associations between perceived support and PTG domains in daily recovery experiences. The present findings corroborate and build on this limited evidence, reinforcing the broader personal, social, and community resources that better explain positive psychological outcomes than abstinence alone. Recovery capital was also strongly associated with psychological flexibility, supporting previous work that links social support, stable housing, and purposeful activity to greater emotional adaptability and value-driven behavior (Bunaciu et al., 2024; Sinclair et al., 2024). Together, these results emphasize that expansive recovery frameworks grounded in recovery capital may provide a more effective foundation for psychological flexibility and growth than abstinence-based models.

Importantly, psychological flexibility was associated with posttraumatic growth and statistically accounted for part of the recovery capital–growth relationship. This extends prior findings that facets of flexibility are related to positive outcomes among trauma survivors (Landi et al., 2022) and aligns with research identifying flexibility as a therapeutic process in acceptance- and commitment-based interventions for substance use disorder populations (Hogan et al., 2025; Krotter et al., 2024). Individuals in recovery may be more likely to experience posttraumatic growth when they demonstrate greater flexibility in handling distressing experiences. These findings therefore highlight psychological flexibility not merely as an outcome, but as a process mechanism through which recovery resources may foster growth.

### 4.1. Limitations

Several limitations should be considered when interpreting the results. First, the cross-sectional design precludes strong causal claims. While we used a mediation framework, indirect effects observed at one time point cannot establish temporal ordering (Maxwell et al., 2011; Weems, 2025). It remains possible that psychological flexibility predicts recovery capital, or that the relationship is bidirectional. These results are therefore best interpreted as evidence of statistical associations that inform future longitudinal hypothesis testing, not mechanisms of change.

Second, all data were based on self-reports, which introduces potential biases such as socially desirable responding (Gorfinkel et al., 2024; King, 2024); such biases have been shown to covary with substance-related harms (Bharat et al., 2023). This reliance may be particularly relevant in recovery research, where participants may underreport substance use or overreport outcomes.

Third, the sample was not demographically representative. Most participants identified as White and binary-gendered, and younger adults were underrepresented. This limitation is consistent with broader gaps in recovery research that under-sample marginalized communities (Lu et al., 2025), and it constrains the generalizability of the observed associations across cultural, racial/ethnic, and gender groups.

Fourth, our simplified model did not adjust for several background factors (e.g., treatment history, co-occurring mental-health conditions, trauma severity). Prior work suggests that these factors may influence both recovery resources and posttraumatic outcomes (Back et al., 2024), and their omission may have introduced uncontrolled confounding.

### 4.2. Future Directions

These findings present several productive avenues for future research. Most critically, longitudinal and experimental designs are needed to evaluate how daily flexibility processes (e.g., mindfulness, values-based action) interact with different domains of recovery capital over time. Such designs would permit temporal ordering and move beyond the associations. Incorporating longitudinal assessment (e.g., baseline, 2-week, 3-month, and 6-month follow-up post-treatment) would strengthen validity by capturing trajectories of change relative to each participant’s baseline.

Future work should also examine recovery capital at the domain level, testing whether specific subscales (e.g., social support, psychological health, coping skills) show distinct associations with psychological flexibility and posttraumatic growth. Expanding sample diversity is equally important; intentional recruitment across diverse cultural, racial/ethnic, and gender groups—through treatment programs, mutual-aid groups, and community partners. Diverse engagement will help capture variability in how recovery capital and psychological flexibility operate across contexts. Finally, future models should test whether the observed indirect effects remain robust when accounting for covariates such as treatment history, co-occurring mental-health conditions, and trauma severity, and should examine whether the pathways differ across subgroups defined by these factors.

An additional limitation of this study is the predominantly young adult composition of the sample, which remains underrepresented in growth-oriented recovery research. Recovery identity and recovery resources are increasingly studied in collegiate settings (Ashford et al., 2018; Hennessy et al., 2025; Smith et al., 2024). Future work should intentionally recruit more diverse samples to examine subgroup differences (e.g., race/ethnicity, gender identity, and recovery pathways).

### 4.3. Clinical Implications

Finally, these directions carry wider clinical implications. Process-based interventions that strengthen psychological flexibility may help individuals translate recovery resources into growth-related outcomes when coping with trauma. At the systems level, integrating harm-reduction principles into specialty substance use treatment may broaden engagement and support multiple pathways to recovery, potentially strengthening recovery contexts that foster adaptive meaning toward growth (Francis et al., 2025; Ganetsky et al., 2025).

At the individual level, clinicians working with trauma-exposed individuals in recovery may benefit from incorporating acceptance and commitment-based strategies that target psychological flexibility alongside traditional recovery support. The finding that psychological flexibility partially mediated the recovery capital–growth relationship suggests that helping clients develop the capacity to approach rather than avoid distressing internal experiences, build on present-moment focus, and take value-directed action may amplify the benefits of existing recovery resources. Practically, this may involve integrating ACT-based modules into recovery programming or training recovery coaches in flexibility techniques.

Still, the non-significant associations between abstinence status and both psychological flexibility and posttraumatic growth carry implications for treatment planning. These findings suggest that recovery outcomes may be better supported by interventions that build on multidimensional recovery capital (social support, coping skills, community involvement, psychological health) rather than focusing exclusively on substance cessation as a measure of success. Clinicians may consider routinely assessing recovery capital and psychological flexibility as treatment targets to identify modifiable strengths that support sustained well-being and growth.

At the system level, these findings reinforce the importance of recovery-oriented systems of care that provide continued access to resources beyond acute treatment. Programs that connect individuals to peer support, stable housing, meaningful employment, and community engagement may create conditions that not only sustain but foster recovery and the psychological flexibility needed for posttraumatic growth. Training and supervision models for substance use treatment counselors and mental health professionals should emphasize the ongoing assessment and continued cultivation of both recovery capital and psychological flexibility toward aligned treatment targets and goals.

## 5. Conclusions

Despite these limitations, the present study advances recovery theory by integrating three frameworks, recovery capital, psychological flexibility, and posttraumatic growth, into a unified account of adaptive recovery processes. The results suggest that recovery capital is linked to posttraumatic growth both directly and indirectly through psychological flexibility. This conceptual integration moves beyond abstinence-only models, positioning growth and resilience as central outcomes of recovery.

With respect to the study’s three hypotheses, all were supported. Consistent with H1 and H2, recovery capital was positively associated with psychological flexibility and posttraumatic growth, whereas abstinence status was not. Consistent with H3, psychological flexibility statistically accounted for part of the association between recovery capital and posttraumatic growth. For theory, this highlights psychological flexibility as a process through which recovery resources may translate into positive transformation. For practice, it suggests that interventions that enhance recovery capital while cultivating flexibility may support sustained well-being among individuals with trauma and substance use histories. Ultimately, this study offers an early step toward broader models of recovery that frame resilience and growth, not only abstinence, as markers of success. Clinically, this suggests that there is value in routinely assessing recovery capital and psychological flexibility as treatment planning targets alongside substance use severity. Incorporating these assessments into treatment planning may help clinicians identify modifiable resources that support not only sustained recovery but also the capacity for positive psychological change following trauma.

## Figures and Tables

**Table 1:** Demographic and characteristic descriptive statistics (N = 244).

Demographic	n (%)	Characteristic	n (%)
* Gender		Recovery Status	
Male	152 (62.30)	Abstinent	148 (60.66)
Female	88 (36.07)	Non-abstinent	96 (39.34)
Non-binary/Other	4 (1.64)	Recovery Identity	
Race/Ethnicity		Yes	161 (65.98)
White	179 (73.36)	No	83 (34.02)
Black	42 (17.21)	Time in Sobriety	
Asian	18 (7.38)	<1 year	157 (64.34)
Other	5 (2.05)	1–5 years	57 (23.36)
Relationship Status		5+ years	30 (12.30)
Single	75 (30.74)	Drug of Choice	
Partnered	54 (22.13)	Alcohol	113 (46.31)
Married	103 (42.21)	Cannabis	35 (14.34)
Divorced/Widowed	12 (4.92)	Opiates	19 (7.79)
Age, *M* (*SD*)	35.91 (10.32)	Other/Polysubstance	77 (31.56)

Note: Demographic and characteristics for variables of interest. Sample sizes vary due to missing data: gender (n = 241); race (n = 239); relationship status (n = 242). * three participants preferred not to disclose their gender.

**Table 2:** Correlation matrix among primary study variables (N = 244). * *p* < 0.05, ** *p* < 0.01, and *** *p* < 0.001.

Measure	1	2	3	4	5	6	7	8
1. PTGI-X	—							
2. ARC	0.21 ***	—						
3. Abstinence	0.04	0.44 ***	—					
4. PPFI	0.26 ***	0.19 **	0.05	—				
5. Time Since Last Use	−0.09	−0.05	−0.01	−0.01	—			
6. Age	0.03	0.15 *	0.06	0.02	−0.01	—		
7. Recovery Importance	0.43 ***	0.07	0.16 *	0.26 ***	−0.02	−0.05	—	
8. Spirituality Importance	0.47 ***	0.16 *	0.01	0.12	−0.04	0.07	0.38 ***	—
*M*	73.97	15.99	0.61	87.08	2.42	35.91	5.45	4.68
*SD*	30.40	6.00	0.49	11.05	1.59	10.32	1.72	2.16

Note: PTGI-X = Post-Traumatic Growth Inventory-X; ARC = Assessment of Recovery Capital; PPFI = Personalized Psychological Flexibility Index.

**Table 3:** Regression of associations between recovery types, posttraumatic growth, and psychological flexibility.

**Model**	**Variable**	***b***	***SE***	***t***	***p***	**95% *CI***	***R* ^2^**
X → Y							
1a	ARC	1.09	0.31	3.42	<0.001	[0.46, 1.71]	0.046
1b	ARC-SUR	2.56	3.98	0.64	0.521	[−5.30, 10.41]	0.002
1c	ARC	1.24	0.35	3.49	<0.001	[0.53, 1.93]	0.05
	ARC-SUR	−4.09	4.3	−0.94	0.351	[−12.63, 4.46]	0.05
X → M							
2a	ARC	0.34	0.11	2.94	0.001	[0.11, 0.57]	0.035
2b	ARC-SUR	1.14	1.45	0.79	0.431	[−1.71, 3.99]	0.003
2c	ARC	0.37	0.13	2.88	0.001	[0.11, 0.62]	0.036
	ARC-SUR	−0.87	1.59	−0.54	0.591	[−3.99, 2.26]	0.036
Regression of posttraumatic growth and psychological flexibility (controlling for recovery capital).							
	**Variable**	***b***	***SE***	***t***	***p***	**95% *CI***	***R* ^2^**
M → Y							
	ARC	0.87	0.32	2.77	0.006 **	[0.25, 1.50]	0.10
	PPFI	0.63	0.17	3.66	<0.001 ***	[0.29, 0.96]	

** *p* < 0.01, and *** *p* < 0.001. Note: ARC = Assessment of Recovery Capital; PPFI = Personalized Psychological Flexibility Index.

## Data Availability

Research data are not shareable.
